# The complete mitochondrial genome and phylogenetic analyses of *Cathartes melambrotus* (Wetmore 1964) (Aves: Cathartidae)

**DOI:** 10.1080/23802359.2025.2461678

**Published:** 2025-02-06

**Authors:** Robert Driver, Renee Li

**Affiliations:** Department of Molecular Genetics and Microbiology, Duke University, Durham, NC, USA

**Keywords:** Cathartidae, mitogenome, scavenger, *Cathartes melambrotus*

## Abstract

*Cathartes melambrotus* is the largest member of the genus *Cathartes*, and soars over the forested areas of Amazonia in search of carrion. The complete mitochondrial genome of *C. melambrotus* was reported in this study. The 19,232 base pair genome consisted of 16 protein coding genes, 25 tRNAs, two rRNAs, and two control regions. The mitochondrial genome contained the avian ancestral duplicated gene region, with the same rearrangements previously reported in Accipitriformes, Cathartiformes, and Stigiformes. With the publishing of the *C. melambrotus* genome all seven Cathartiformes species mitochondrial genomes are available and can be included in subsequent phylogenetic and genomic analyses.

## Introduction

**Figure 1.**
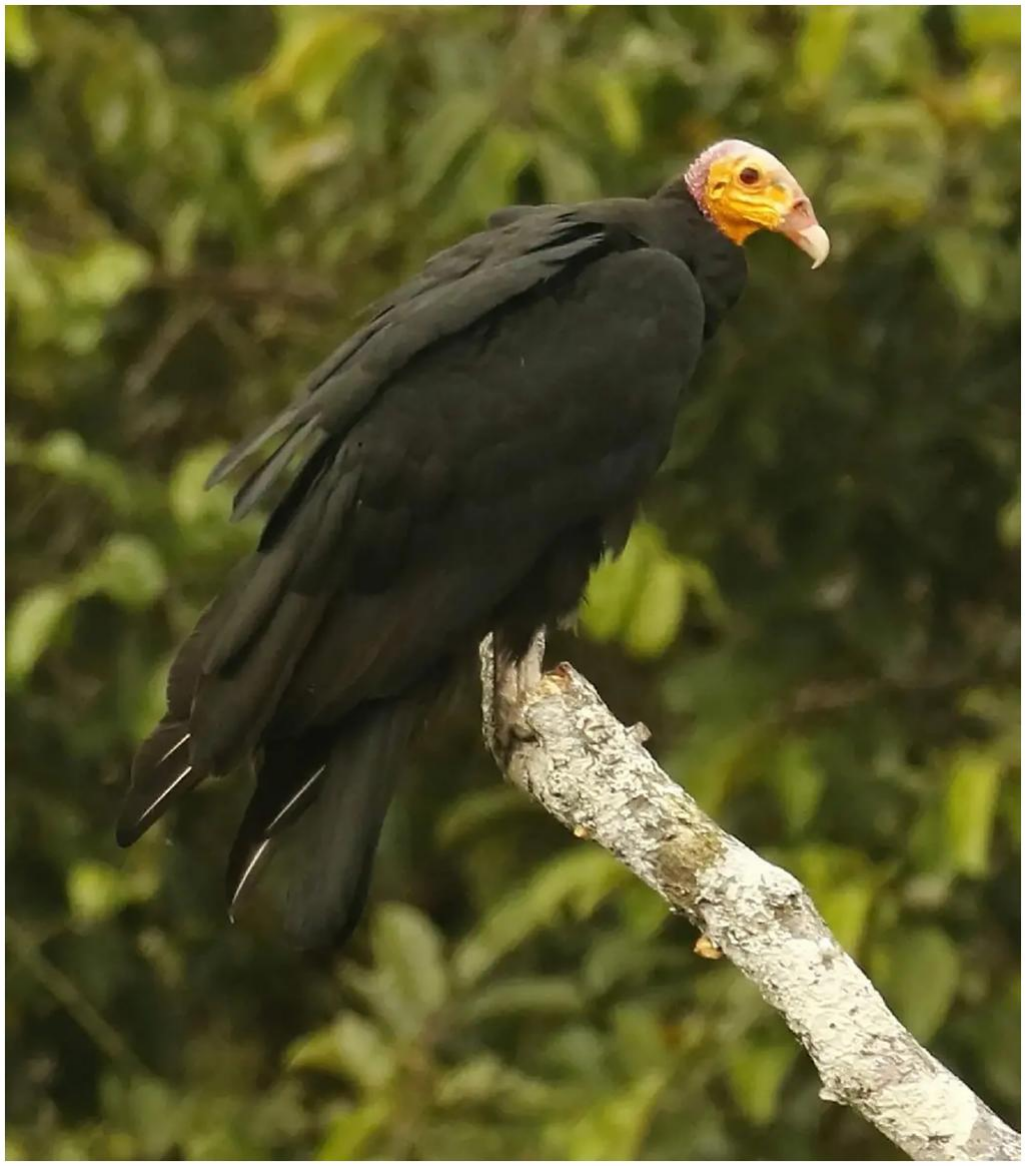
Species image of an adult *C. melambrotus*. The bird is characterized by dusky undersides of the inner primaries, that contrast pale secondaries and outer primaries. This is in contrast to the other species of *Cathartes*, in which all remiges are pale. The plumage is completely black, and darker than *C. aura*. Perched photograph by tony castro, used with creative commons license.

*Cathartes melambrotus* (Wetmore 1964) is a species of vulture in the family Cathartidae, order Cathartiformes (New World vultures). *C. melambrotus* is the largest member of the genus *Cathartes* with black plumage and a distinctive featherless head in varying shades of red, yellow, and blue. It can be distinguished from its congeners *C. burrovianus* and *C. aura* by the dark gray color of the undersides of the inner primaries, in contrast to the pale gray undersides of all primaries in the other *Cathartes* species (Figure 1; Schulenberg et al. 2010). *Cathartes melambrotus* are found almost exclusively in dense neotropical rainforest with a range mostly restricted to the Amazon basin (Jones 2020). Due in part to its relatively inaccessible habitat, *C. melambrotus* is perhaps the least known member of the seven species of Cathartidae, first being described only in the latter half of the twentieth century (Wetmore 1964). There is no complete nuclear or mitochondrial genome available for *C. melambrotus*.

Our study presents the first complete mitochondrial sequences of *C. melambrotus*. From this sequence, we were able to build the first phylogenetic tree investigating the relationships of Cathartidae using the mitochondrial genomes of all seven species. The *C. melambrotus* mitochondrial genome will enable future research to analyze all seven extant Cathartidae species for systematic relationships, genomic investigations, and potential functional studies.

## Materials and methods

A specimen of *Cathartes melambrotus* was shot by Sidnei Dantas on November 9^th^ 2015, in terra firme forest habitat at the Ferreira Penna Scientific Station in the Caxiuanã National Forest in the Melgaço municipality in the state of Pará, Brazil (-1.7375° S, −51.4555° W). At the time of collection, the bird’s mass was 1.47 kg. The specimen’s iris was red, the tarsi grayish pink, and the maxilla and mandible were pink. The soft part colors are inconsistent with *C. aura*, and the mass greatly exceeds *C. burrovianus* (Eitniear 2020), identifying the specimen as *C. melambrotus*. The skull was fully ossified, and there was fur in the stomach. The bird was a male, with testes measuring 8 × 4 mm. The bird’s muscle, heart, and liver tissues were sampled and inventoried by José Nílton da Silva Santa-Brígida, and a tissue sample was deposited at the Academy of Natural Sciences of Philadelphia (https://www.ansp.org). A sample of the tissue was provided by Dr. Nathan H. Rice (rice@ansp.org) under the tissue specimen voucher ANSP #31121. The associated bird specimen is vouchered at the Museu Paraense Emílio Goeldi in Belém, Brazil under the original preparator number CAX-15-013.

**Figure 2.**
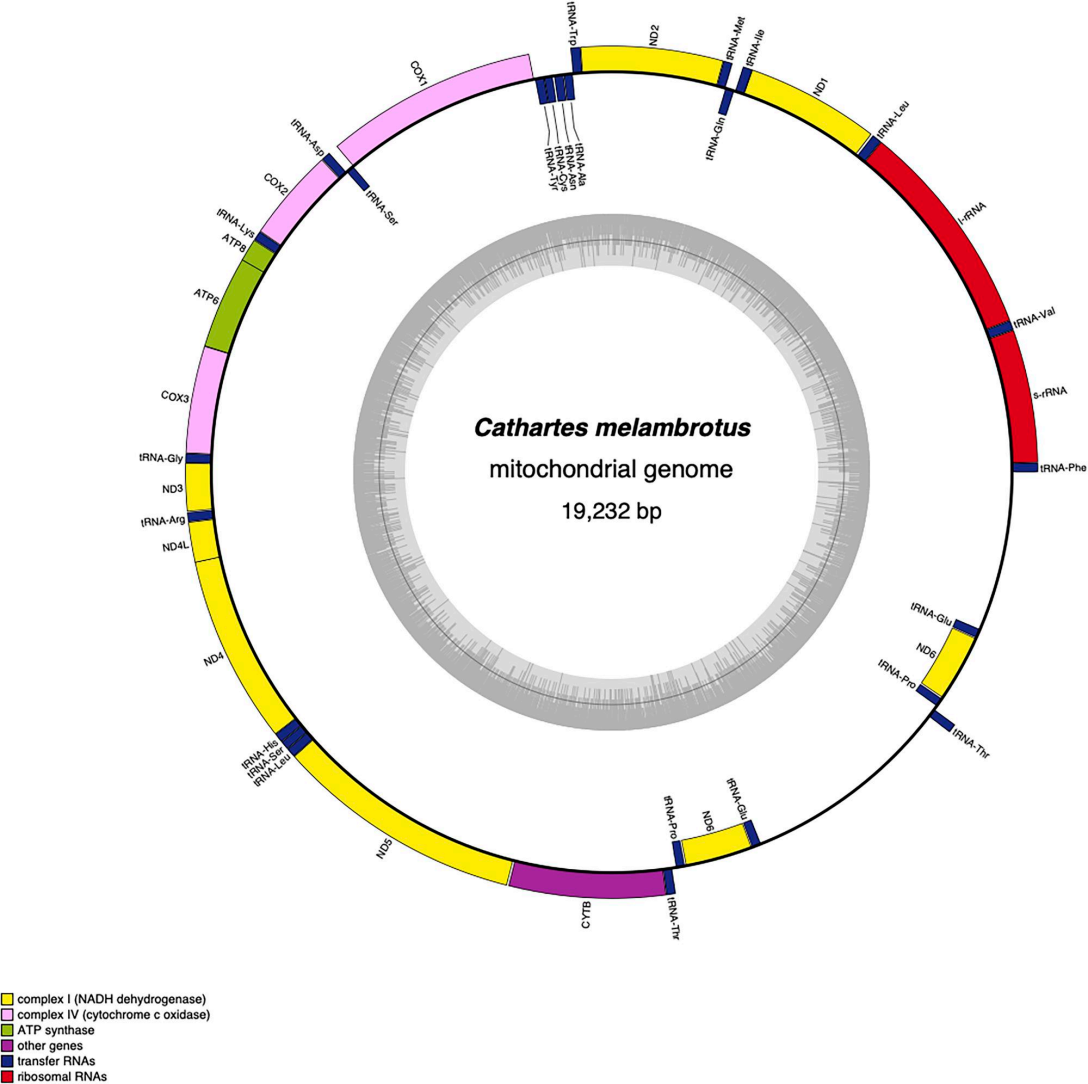
The circular complete mitochondrial genome map of *C. melambrotus*. This map was created with the annotation software MitoZ, and displays mitochondrial genomic features and their strand placement in the assembly. In the outer circle coding genes are displayed in green, tRNAs in red, rRNAs in orange, with two control regions. Sequences displayed within the circle represent genomic features located on the heavy strand, where as genomic features displayed outside of the circle are located on the light strand. In total, 16 protein coding genes, 25 tRNAs, and two rRNAs were present. There are two copies of the control region, nad6, and tRNA-Thr, tRNA-Pro, and tRNA-Glu, indicative of the duplicated rearrangement of this region in this particular group of birds. The inner circle displays the depth of coverage for each position in them mitochondrial genome.

*Cathartes melambrotus* samples were shipped in ethanol. DNA extraction, sequencing, and initial data analyses were performed by CD Genomics (www.cd-genomics.com, Shirley, New York, USA). We used DNeasy Blood & Tissue Kit (Qiagen, Cat. No.: 69504) for mitochodrial DNA extraction. Sequencing libraries were generated using NEBNextR UltraTM DNA Library Prep Kit for Illumina (NEB, USA) and NEBNext^®^ Multiplex Oligos for Illumina^®^ (96 Index Primers, more information about the primers can be found in the supplementary materials, Primers.docx). The mitochondrial genome was sequenced using Illumina NovaSeq6000 PE150 platform (NovaSeq 6000 S4 Flow Cell, NovaSeq 6000 S4 Cluster Cartridge, NovaSeq 6000 S4 SBS Cartridge, and NovaSeq 6000 S4 Buffer Cartridge were used). We mapped exact reads to the reference mitochondrial genome, in this case *Cathartes aura*, assembled the reads using Unicycler, and annotated the structure using the annotate subcommand of MitoZ (Meng et al. 2019). We produced a circular map of the mitochondrial genome using OGDRAW (Figure 2; Greiner et al. 2019).

For phylogenetic analysis, we took advantage of complete mitochondrial genome availability of all six other Cathartid species. We aligned our *C. melambrotus* sequence with the complete mitochondrial genomes of *C. aura* (Slack et al. 2007, GenBank Accession #AY463690.1), *C. burrovianus* (Urantówka et al. 2021, GenBank Accession #MN720441.1), *Coragyps atratus* (Urantówka et al. 2021, GenBank Accession #MN720440.1), *Sarcoramphus papa* (Urantówka et al. 2021, GenBank Accession #MN720442.1), *Gymnogyps californianus* (De Panis et al. 2021, GenBank Accession #BK059163.1), and *Vultur gryphus* (Urantówka et al. 2021, GenBank Accession #MN720444.1). We also included the complete mitochondrial genome of *Sagittarius serpentarius* (Mahmood et al. 2014, GenBank Accession #NC_023788.1) as an Accipitriformes outgroup. We aligned complete mitochondrial sequences using the E-INS-I default parameters in MAFFT (Katoh and Standley 2013). Following alignment, we ran a maximum-likelihood tree using IQ-TREE with automatic model selection and 1000 Shimodaira-Hasegawa-like approximate likelihood ratio test replicates (Minh et al. 2020). We rooted the tree using the *Sagittarius serpentarius* branch and visualized using iTOL software and IcyTree (Vaughan 2017; Letunic and Bork 2019).

## Results

The complete mitochondrial genome of *C. melambrotus* had a total length of 19,232 base pairs and was deposited with the accession number #PQ153910 in the GenBank database (Figure 2). The mitogenome contains 16 protein coding genes, 25 tRNAs, two rRNAs, and two control regions. In contrast to the standard avian gene order (Desjardins and Morais 1990; McLaughlin et al. 2023), the *C. melambrotus* mitochondrial genome has two copies of the control region, nad6 gene, and tRNAs tRNA-Thr, tRNA-Pro, and tRNA-Glu. The overall base-composition of this mitogenome was 31.68% guanine, 30.53% thymine, 23.76% adenine, and 14.04% cytosine. The AT and GC content of the complete mitogenome was 54.28% and 45.72%, respectively. Average depth of coverage was 7,744x across the mitogenome (Figure S1).

**Figure 3.**
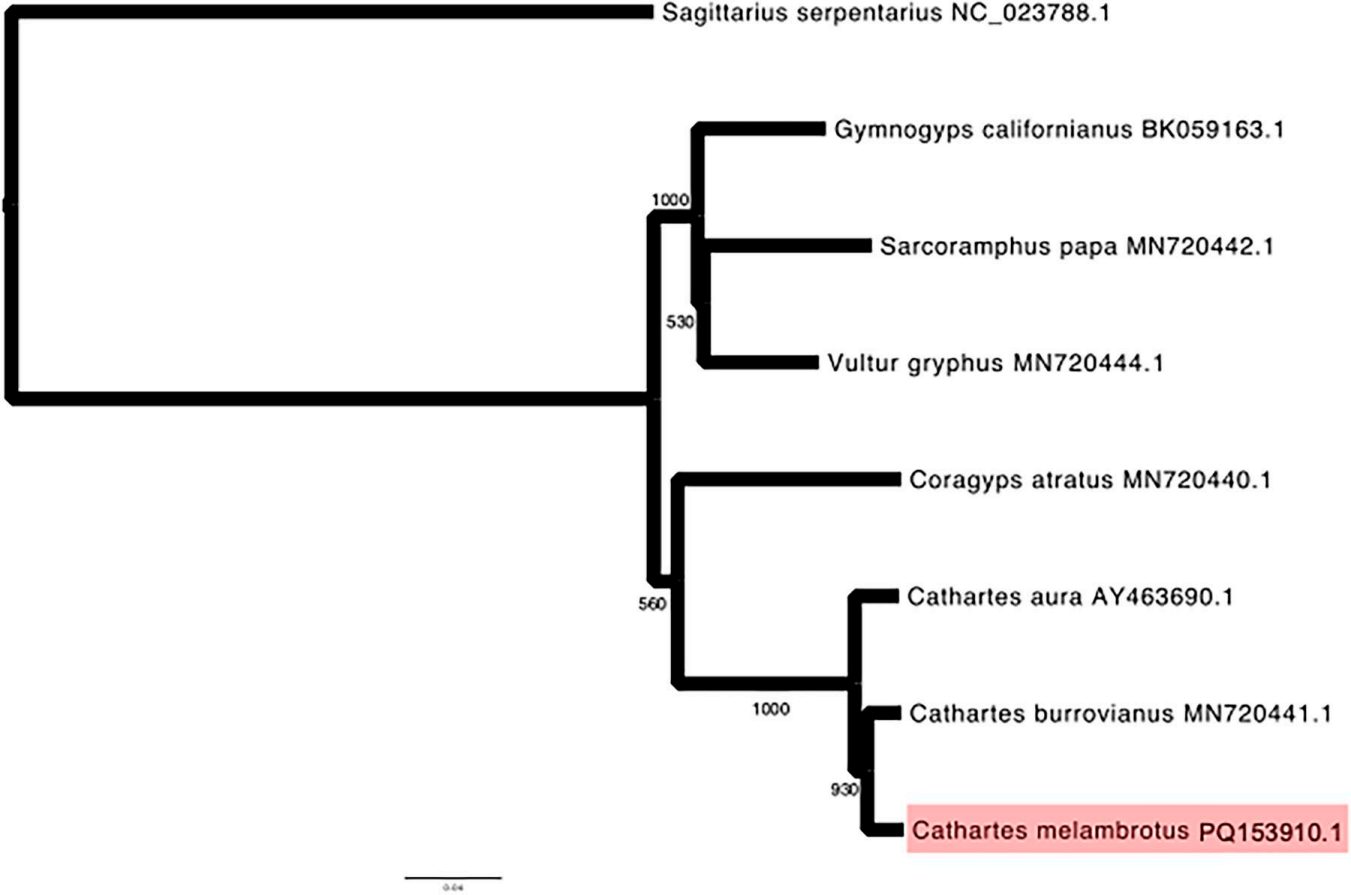
Maximum-likelihood phylogenetic inference with the mitogenomes of all species of Cathartid vultures. The bootstrap support values are displayed on branches to show support from 1,000 bootstrap replicates. In addition to *C. melambrotus* (GenBank accession #PQ153910.1, highlighted in red), the following sequences were retrieved from GenBank and used: *C. aura* (Slack et al. 2007, GenBank accession #AY463690.1), *C. burrovianus* (Urantówka et al. 2021, GenBank accession #MN720441.1), *Coragyps atratus* (Urantówka et al. 2021, GenBank accession #MN720440.1), *Sarcoramphus papa* (Urantówka et al. 2021, GenBank accession #MN720442.1), *Gymnogyps californianus* (De Panis et al. 2021, GenBank accession #BK059163.1), and *Vultur gryphus* (Urantówka et al. 2021, GenBank accession #MN720444.1). We also used the complete mitochondrial genome of *Sagittarius serpentarius* (Mahmood et al. 2014, GenBank accession #NC_023788.1) as an Accipitriformes bird of prey outgroup.

We constructed a phylogeny of all seven species of New World vultures (Family Cathartidae) using the complete mitochondrial genomes of each species. The phylogenetic tree clearly separates all vulture species, with two distinct clades (Figure 3). One clade consisted of the three *Cathartes* species as well as *Coragyps* (Figure 3). In this clade, *C. melambrotus* grouped with *C. burrovianus*, with *C. aura* sister to the other two *Cathartes* species. *Coragyps* was then sister to the *Cathartes* genus. The other clade consisted of the remaining three Cathartidae species.

## Discussion and conclusion

In this first sequencing of the complete mitochondrial genome for *Cathartes melambrotus*, we report duplications of the control region, nad6, and three tRNA genes: tRNA-Thr, tRNA-Pro, and tRNA-Glu. This duplication and associated rearrangements are consistent with mitochondrial genome sequences from other Cathartiformes and Accipitriformes species, as well as Strigiformes, suggesting that the duplication and rearrangements in its present order potentially occurred in the ancestral lineage of all modern hawks, vultures, and owls (Hanna et al. 2017; Urantówka et al. 2021). In our phylogeny using complete mitochondrial genomes, the seven Cathartidae species grouped into two distinct clades. The clade consisting of the three *Cathartes* species and *Coragyps* had a topology identical to reports from nuclear markers (Johnson et al. 2016), but does not agree with a previously published topology using only the cytb gene (Ericson 2012).

With all seven species of Cathartidae now with complete mitochondrial genomic data, future research can build phylogenies or compare mitochondrial evolutionary history between all species in the family. Mapping the complete mitochondrial genome of *C. melambrotus* is an important stride for researching this understudied and remote species.

## Supplementary Material

FigS1_depth_new.jpeg

Primers used for sequencing.docx

## Data Availability

The genome sequence data that support the findings of this study are openly available in GenBank of NCBI at [https://www.ncbi.nlm.nih.gov] under the accession no. PQ153910. The associated BioProject, SRA, and Bio-Sample numbers are PRJNA1167675, SRS22791288, and SAMN44010771 respectively.
