# Supplementary figures and images for: The complete mitochondrial genome and phylogenetic analyses of Cathartes melambrotus (Wetmore 1964) (Aves: Cathartidae)
Source: Mitochondrial DNA B Resour. 2025 Feb 6;10(3):187–91. doi: 10.1080/23802359.2025.2461678 (PMC11803758; doi:10.1080/23802359.2025.2461678)

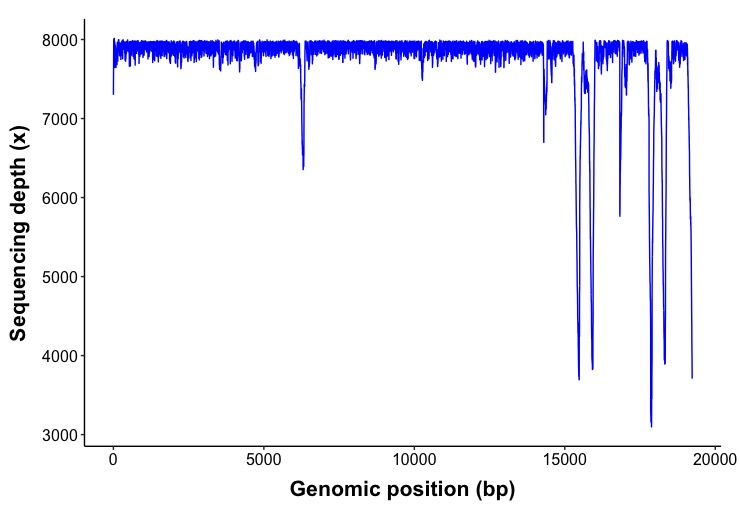

Supplement: FigS1_depth_new.jpeg [file TMDN_A_2461678_SM3571.jpeg]
