# Supplementary material for: The complete mitochondrial genome and phylogenetic analyses of Cathartes melambrotus (Wetmore 1964) (Aves: Cathartidae)
Source: Mitochondrial DNA B Resour. 2025 Feb 6;10(3):187–91. doi: 10.1080/23802359.2025.2461678 (PMC11803758; doi:10.1080/23802359.2025.2461678)
Supplement: Primers used for sequencing.docx [file TMDN_A_2461678_SM3570.docx]

Primers used for sequencing:

The adapter for Illumina:

i5 Primer:
5'-AATGATACGGCGACCACCGAGATCTACAC [i5index] ACACTCTTTCCCTACACGA
CGCTCTTCCGATCT-3'

i7 Primer:
5'-CAAGCAGAAGACGGCATACGAGAT [i7index] GTGACTGGAGTTCAGACGTGTG
CTCTTCCGATCT-3'
